# Flow and performance: a quantitative study of elicitation modeling in a piano performance perspective

**DOI:** 10.3389/fpsyg.2024.1386831

**Published:** 2024-07-15

**Authors:** Junrui Li, Ying Xu, Xiaoyu Wang, Xinran Yuan, Shilin Liu, Meihui Li, Xiubo Ren, Dan Yang, Siqi Liu, Zhenxu Jiang, Qiaoqiao Gou, Miaomiao Yang, Qiujian Xu

**Affiliations:** ^1^Institute for Artistic Cognition and Brain Science, Department of Music, School of Arts and Design, Yanshan University, Qinhuangdao, China; ^2^YSU & DCU Joint Research Centre for the Arts, Music College, Daegu Catholic University, Daegu, Republic of Korea

**Keywords:** flow experience, dynamic balance of challenges and skills, performance, Shannon entropy, quantification of musical features

## Abstract

**Introduction:**

Flow, defined as a heightened state of consciousness characterized by intense concentration during an activity, is influenced primarily by the perceived challenge and the dynamic equilibrium of skills. This investigation focuses on the patterns of flow state attainment and its elicitation mechanisms within the context of piano performance among Chinese music college students.

**Methods:**

Our study establishes a framework for accessing flow, utilizing quantitative data from music ontology to gauge the level of challenge and the level of music acquisition to assess skills. Additionally, we integrate external factors such as music culture heterogeneity and demographic variables to elucidate the causes and moderating effects of flow on piano performance.

**Results:**

The findings reveal a positive correlation between flow and performance, with the model of challenge and skill induction partially explaining these results. Notably, melodic Shannon Entropy emerges as a potential indicator of challenge, suggesting its relevance in future studies on flow.

**Discussion:**

This research provides multidimensional insights into the interplay between performance and flow in piano performance, guiding future investigations to explore the musical quantitative perspective more deeply.

## 1 Introduction

### 1.1 Flow in piano performance

Flow, a concept extensively explored across various disciplines such as sports (Kim and Ko, 2019; Goddard et al., 2023), dance (Łucznik and May, 2021), games (Pham et al., 2022), and chess (Tozman et al., 2017), has garnered multidisciplinary attention. In the field of music, particularly in the realm of music performance (O’Neill, 1999; Spahn et al., 2021; Antonini Philippe et al., 2022), music listening (Diaz, 2013; Karmonik et al., 2016; Loepthien and Leipold, 2022), music composition (MacDonald et al., 2006; Chirico et al., 2015), and music education (Custodero, 2002; Bernard, 2009), exhibit a pronounced inclination toward flow. Notably, musical performance situations are the most conducive to flow experiences. Moral-Bofill et al. (2023) contends that classical musicians, due to their academic and structured learning environments, frequently experience heightened states of flow. The suggestion is made that musical performance or recital serves as an effective activity for inducing flow experiences. Moreover, music students, alongside elite classical musicians, demonstrate a proclivity for experiencing flow. Empirical investigations indicate divergent trends and patterns of flow scores among student musicians compared to their professional and amateur counterparts, necessitating targeted independent research. In the realm of musical performance, theoretical advancements in understanding flow, particularly in piano performance activities, are still evolving (De Manzano et al., 2010). Previous studies have explored flow across various music performance categories, including violin, piano, woodwinds, and voice. Surprisingly, results revealed that the piano had a significantly lower flow score (De Manzano et al., 2010). However, Bakker (2005) contradicts this, asserting that piano is the most flow-inducing musical activity. This discrepancy prompted the focus of our study on the distinct research direction of piano performance, aiming to investigate the patterns of flow state attainment among music undergraduates in piano performance situations.

Moreover, our study aims to elucidate the mechanisms triggering this phenomenon. Within the context of music performance, an individual’s skills inherently contribute to predicting and influencing flow, while external challenges exert a relative impact on the flow process. Previous research has attempted to observe and analyze flow by predefining skills and challenges, considering them as indicators of the flow experience (Haumann et al., 2018). Fullagar et al. (2013) specifically investigated the state of flow among music major students by examining the interplay between the perceived challenge level of a musical piece and the skills required for its performance. Currently, this variable remains the most prevalent and crucial premise influencing the flow experience. However, its context specificity and susceptibility to the interaction of other unidentified factors underscore the need for further in-depth exploration in subsequent research.

In explicating the attainment of a flow state within the context of piano performance, it is imperative to consider numerous external factors alongside the aforementioned internal ones. Among these, music-related factors emerge prominently. This study focuses on specific aspects of interest, namely (1) practice duration, where musicians undergoing prolonged and high-quality practice sessions are posited to be more predisposed to entering a state of flow (Tan et al., 2021), with practice duration identified as a predictor of flow in this population (Marin and Bhattacharya, 2013). Waite and Diaz (2012) observed a similar phenomenon in the population of undergraduate music students. The explanation for this phenomenon is closely tied to an individual’s perceived equilibrium between challenge and skill states, where the difficulty of challenges presented by various musical materials dynamically influences an individual’s skill. (2) Musical characteristics: Some studies employ designated or specific musical compositions to predict various conditions under which flow occurs, indicating differences in flow associated with different musical composers. A recent study provided evidence from a neurological perspective, in which HRV (heart rate variability) was collected from 22 conservatory piano certified pianists during the performance of 2 assigned pieces and 1 self-selected piece, and performer heart rate data were taken using a polar watch for 5 min before, during, and 5 min after the performance, and flow state was measured. The results showed that for professionally assessed piano performers, they produced the highest level of flow experience during the performance of Bach’s works (Jha et al., 2022); (3) Musical styles: Jazz/contemporary and traditional musicians exhibit higher flow state scores compared to classical traditional musicians. Piano subjects report experiencing increased flow when playing certain musical styles, with the Romantic style identified as particularly likely to induce flow (Marin and Bhattacharya, 2013). (4) Type of performance: The category of instrument played leads to variations in the flow experience. Cohen and Bodner (2021) found a positive correlation in the flow experience of professional orchestral musicians, though acknowledging a lack of validation studies and the potential for spurious effects. (5) Musical cultural heterogeneity: Exploring the intersection of musical correlates with cultural identity offers potential evidence for musical cultural heterogeneity in flow states. Musical culture encompasses the informal acquisition of musical knowledge in diverse cultural environments (Wang et al., 2024), significantly influencing aspects of music perception, such as melodic expectations (Demorest et al., 2016). China-specific cultural heterogeneity similarly affects the cross-cultural nature of the flow experience (Zhou and Luo, 2012). However, the majority of publications in the field hailed from Western cultural contexts, such as the United States (number of publications is 30), the United Kingdom (12), and Australia (10), with limited attention directed toward mobility research in Eastern cultures, especially within Chinese cultural contexts.

Another noteworthy external consideration that has garnered attention pertains to demographic factors, encompassing variables such as gender, age, and other demographics. Nevertheless, the exploration of these factors in relation to flow in the realm of music has yielded inconclusive results. While some studies propose that certain demographic factors are irrelevant to musical flow (Sinnamon et al., 2012; Marin and Bhattacharya, 2013; Wrigley and Emmerson, 2013), others validate the relevance of certain factors to the experience of flow. Notably, age has been identified as a significant predictor of flow, with recent research establishing a high correlation between a musician’s age and years of performing experience. Conversely, findings regarding gender-based differences in flow experiences have not demonstrated consistency across studies (Moral-Bofill et al., 2019). Consequently, the inclusion of pertinent demographic factors was deemed imperative in this study (see Barthelmäs and Keller, 2021).

Previous research in this domain has employed psychophysiological metrics for correlation studies, utilizing measures such as questionnaires (Araújo and Hein, 2019 Kim and Ko, 2019), EEG data (Katahira et al., 2018 Tan et al., 2023), and heart rate variability data (Jha et al., 2022). The concept of the ‘flow state’ in music entails a technique for performing musical material inseparable from the music itself. However, the dynamics of the flow experience in piano performance have not been characterized in terms of their relation to musical ontology in several exploratory cross-sectional studies. Musical ontological characteristics encompass parameters such as technique (the level of technical performance of the musical material), rhythm (the alternation of sounds and pauses), sound quality (the quality characteristics of the musical material), and musicality (musicianship). To comprehensively interpret the musical experience, considerations extend beyond these four aspects, encompassing information about the instrument being played, the acoustics of the music, and the effects produced by the musical sound. Zielke et al. (2023) conducted two correlation studies analyzing specific musical characteristics corresponding to flow by prompting musicians to recall information about the timing of flow initiation and interruption during performance. Results revealed correlations between flow-inducing and interrupting timing, pitch, and timbral dimensions. Subsequent in-depth analyses in their continuation study focused on the scores and melodies played by participants, uncovering common features of flow states such as gradual movement, repetition, and the absence of detached movement in melodies. These findings tentatively explicate the interconnectedness of flow and musical ontological features, setting the stage for our exploration of the correlation between piano performance and flow, particularly in terms of extracting and quantifying musical ontological features.

In the context of this study, musical ontological characteristics will serve as a metric for assessing the challenge level. Musicians face the imperative task of selecting repertoire that aligns with their skill proficiency. A discrepancy between challenge and skill goals can lead to manifestations of anxiety and boredom. Consequently, participants are encouraged to choose repertoire that not only aligns with their skill level but also presents a genuine challenge (Kirchner, 2011). The Shannon Entropy algorithm emerges as a promising tool for extracting information about musical complexity and quantifying the challenge level (Gündüz, 2023). This algorithm facilitates the derivation of musical information embedded in a piano performance, along with its complexity level, thereby enabling its integration into a quantitative analysis within a challenge and skill balance model.

Therefore, we pose the initial research question: What patterns and mechanisms lead to a state of flow in Chinese piano students who are in a performance situation? To address this question, we will construct a framework for accessing the flow state based on the challenge level represented by quantitative data from music ontology and the skill level represented by the level of music acquisition. Furthermore, we aim to elucidate the underlying causes by considering external factors such as music culture heterogeneity and demographic variables (music culture heterogeneity, demographic factors, etc.).

### 1.2 The correlation between flow and performance in the piano performance perspective

Flow, characterized as an optimal experiential state, serves various social functions, notably contributing to performance enhancement (De Manzano et al., 2010). In the realm of music performance, flow emerges as a theoretical framework intrinsic to learning behavior, enhancing an individual’s capacity to confront challenges and accelerate skill acquisition as musical education advances with increased practice and deliberate engagement (Palomäki et al., 2021). Within a flow experience, piano performers attain an optimal state, resulting in peak performance levels (Palomäki et al., 2021). The intermittent experience of flow motivates music learners to sustain and advance their professional skills, with research indicating a positive correlation between flow and achievement among young music students (O’Neill, 1999), as well as successful performances reported by music students and professionals (Clark et al., 2014), with the challenge and skill balance profile explaining this phenomenon. Musicians aspire to maintain a state of flow in various contexts, including performance, competitions, or exams. Fullagar et al. (2013) explored the relationship between student musicians’ flow experiences and music performance anxiety within a challenge and skills framework, utilizing the complexity of music performance as an indicator of challenge in relation to the perceived skills required. This study provides a referenceable research framework for our focus on the relationship between flow and performance.

The choice of context for investigating performance is crucial. Even under conditions of challenge and skill balance, changes in task types can trigger different flow outcomes. Research suggests a link between flow and classical music students’ performance during exams (Clark et al., 2014; Cohen and Bodner, 2019). An analogous link is also observed in the context of musical performance (Spahn et al., 2021). Bullerjahn et al. (2020) integrated these contexts and conducted a cross-sectional survey paradigm with questionnaires in a music competition environment. They analyzed the interrelationships between practice time and motivational factors among participants in different competition categories. The results revealed that challenge was the most significant motivational factor for all participants, particularly for classical soloists. Furthermore, they noted that the significance of the music competition environment for participants’ musical ability and development arises from the interaction between intrinsic motivation and the determination to practice. Therefore, we posit that the competition context established in this study may serve as an effective experimental setting.

Therefore, we pose the second research question: What is the role of the attained state of flow among Chinese music college students in moderating their piano performance, particularly in an examination situation where piano performance constitutes the primary content?

## 2 Method

### 2.1 Participants

Participants for this study were recruited through the public campus service platform of Chinese universities and colleges. Inclusion criteria comprised individuals who (1) had a minimum of 3 years of musical training experience, (2) had undergone at least one musical performance, and (3) were 18 years of age or older. A total of 109 music college students (major in piano) were screened to participate in the experiment, and the participants were mainly distributed in the first year of college, accounting for 27.5% (*N* = 30) and the third year of college, accounting for 25.1% (*N* = 28) (refer to Table 1 for specifics). There was a larger proportion of females (*N* = 78, 71.6%), and their ages ranged from 18 to 22 years old, with a mean age of 19.51 years. The results of the sample’s diversity distribution in the self-assessed music-related elements were as follows: in terms of music acquisition, the vast majority of the participants had received 10–13 years of music education up to this point in time; in addition, more than 80% of the participants had mastered basic music skills such as fundamental knowledge of music theory and solfeggio; and with regard to subjectively-assessed musical ability, the majority of the participants perceived themselves as being at an intermediate level (Ave = 3.43, 45%, *N* = 51).

**Table 1:** Descriptive statistics of basic information.

Variable	Categories	*N*	Percentage %
Gender	Male	31	28.4
	Female	78	71.6
Age	18	25	22.9
	19	36	33.0
	20	22	20.2
	21	19	17.4
	22	7	6.4
Grade	Freshman	30	27.5
	Sophomore	35	32.1
	Junior student	28	25.7
	Senior student	16	14.7
Years of piano study	1–3	10	9.2
	4–6	27	24.8
	7–9	23	21.1
	10–13	26	23.9
	14–16	14	12.8
	17–19	0	0.0
	20 and up	9	8.3
Piano training time per day	In 30 min	8	7.3
	30 min - 1 h	7	6.4
	1–1.5 h	23	21.1
	1.5–2 h	20	18.3
	2–3 h	32	29.4
	4–6 h	13	11.9
	6 h and up	6	5.5
Musical skills acquired	Vocal performance	44	40.4
	Instrumental performance	101	92.7
	Music appreciation	61	56.0
	Harmony theory knowledge	57	52.3
	Music score readability	90	82.6
	Solfeggio	92	84.4
	Music composition	23	21.1
	Music history knowledge	48	44.0
	Fundamental knowledge of music theory	88	80.7
Musical ability	1	4	3.7
	2	7	6.4
	3	51	46.8
	4	32	29.4
	5	15	13.8

### 2.2 Challenges and skills models

Primarily, a database of piano pieces was curated by a specialized research team with expertise in piano performance examinations. To mitigate the influence of extraneous factors and minimize objective variations, the following criteria were established: (1) the piano music collection encompasses both Chinese and Western piano compositions; (2) it spans diverse periods, composers, and genres. Adhering to these criteria, the Experimental Piano Collection comprises 100 piano works. Of these, 70 are Western piano compositions, spanning the Romantic, Classical, and Baroque periods, while the remaining 30 are Chinese piano works composed between 1934 and 2003.

Shannon Entropy is one of the concepts in information theory, which is used to measure the uncertainty and randomness of a piece of information, and has multiple meanings in physics, psychology and musicology. In cognitive psychology, the Shannon Entropy can be used to analyze the way people process information and their cognitive load in complex situations, such as information processing and decision-making behaviors; and in music, the Shannon Entropy has been used to measure the complexity of a musical piece and to differentiate between musical genres (Febres and Jaffe, 2017 Gündüz, 2023).

In this study, we use the Shannon Entropy (later referred to as SE) as a measure of challenge in piano works. The reason is that in piano performance, individuals are not able to unify the definition of difficulty and challenge for any type of work, so the number of notes in the piano score all over the piano becomes an objective measure of challenge, and the notes contained in the score are not subjective to the performer’s subjective influence of the challenge, so the study selects the SE as one of the measures of musical complexity. Following Gündüz (2023), we randomly selected six musical compositions for the calculation of the SE based on the criteria of piano performance and cultural identity of the work (See formula below). Specifically, we include the notes contained in the score (except for ornamental notes that do not account for time values) in the data pool for calculation and define them as the Entropy of the piece. To refine the findings, we will also provide the Entropy of the entire piece separately (Shannon Entropy _overall_, SE), the soprano voice (Shannon Entropy _right hand_, RSE), and bass voice (Shannon Entropy _left hand_, LSE) values (see Equation 1).


(1) $$S\left(P\right)=-\frac{1}{\ln2}\frac{1}{n_{i}}\left[n_{ic4}\ln n_{ic4}+n_{\mathrm{id}4}\ln n_{\mathrm{id}4}+n_{ie4}\ln n_{ie4}-n_{i}\ln n_{i}\right]$$

The data included in the skill model were obtained from the questionnaire CFSS-2 and were categorized into four dimensions: (1) years of piano study; (2) Average hours of piano practice per day; (3) Acquired musical skills; and (4) self-assessment of musical ability. The equilibrium model value for the challenge and skill was computed as the absolute difference between challenge (entropy of works) and skill (four dimensions) (Fullagar et al., 2013).

### 2.3 Measurements

#### 2.3.1 Demographic information

This self-administered questionnaire serves as a tool for gathering fundamental information about the participants. Comprising eight questions, the survey requires approximately 02:00 min for completion and encompasses essential details, including the subject’s age, gender, and academic grade. Moreover, it incorporates musical information related to the subject’s years of musical training, acquired musical skills, average daily duration dedicated to musical training, and a self-assessment of their musical proficiency.

#### 2.3.2 Flow state scale-2

The Flow State Scale-2 (FSS-2, Jackson et al., 2004) serves as a self-report instrument for quantifying flow intensity through a 36-item, nine-dimensional scale, with each dimension consisting of four question items. This scale, widely employed in the field of music performance, requires participants to reflect on the recently completed activity while responding to questions. Subjects express their agreement on a 5-point Likert scale ranging from 1 (strongly disagree) to 5 (strongly agree). To minimize disruption to participants, the scale is administered within 1 h of the test conclusion (Cronbach’s alpha = 0.924) (Jackson and Eklund, 2002). Mean scores are computed for each of the nine flow dimensions, each comprising 3–4 question items, and an overall mean flow score is determined. For the purposes of this study, flow scale scores are categorized into three groups: low agreement (mean scores ranging from 1 to 2), indicating a lack of inherent flow experience; moderate (mean scores ranging from 2 to 4), suggesting some endorsement of the flow experience; and high (mean scores ranging from 4 to 5), signifying strong agreement with the flow experience in the chosen activity. The FSS-2 exhibits sufficient reliability, construct validity, and internal consistency, establishing it as an excellent measure of state flow experience.

#### 2.3.3 Dispositional flow scale–2

The Dispositional Flow Scale-2 (DFS-2, Jackson and Eklund, 2002) evaluates the general inclination to experience flow in a specific situation. Comprising four dimensions, it features 33 question items corresponding to the FSS-2, differing only in wording and phrase tense. Participants respond to the DFS-2 questions by reflecting on their overall experience in the situation across various contexts and timeframes, or their typical experience during activities such as work or leisure. Responses are rated on a 5-point Likert scale, ranging from 1 (never) to 5 (always). It is essential to complete the DFS-2 independently of direct engagement in the activity. This scale was exclusively administered to the English-speaking sample. The global flow score is derived by summing all the question items. The scale demonstrates high reliability, with Cronbach’s alpha measuring at 0.931.

The FSS-2 and DFS-2 underwent translation (resulting in the Chinese versions CFSS-2 and CDFS-2) and cross-cultural validation by Sinology, ensuring a valid measure of the flow experience for Chinese participants (Table 2). Coefficients for the CFSS-2 ranged from 0.64 to 0.92 (M = 0.77), and for the CDFS-2, coefficients were 0.65–0.92 (M = 0.78). Five out of the nine flow factors surpassed 0.7, indicating a considerable level of stability. Consequently, both the CFSS-2 and CDFS-2 demonstrated acceptable reliability and validity, warranting their inclusion as indicators of subjects’ flow status.

**Table 2:** Questionnaire description.

	CFSS-2	CDFS-2
Number of items	45	45
Fill in time	State of the process at the end of the exam	Mobility at the end of exams
Scope of Filling	From 1 (strongly disagree) to 5 (strongly agree)	From 1 (strongly disagree) to 5 (strongly agree)
Mode of filling	Fill in online	Fill in online

### 2.4 Procedure

**Figure 1:**
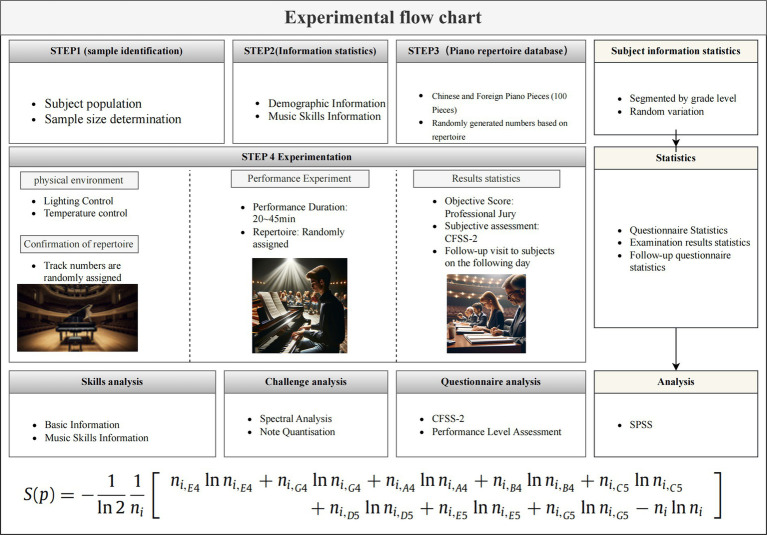
Flow chart of the experiment. Images generated by ChatGPT, version 4.0. The enhancement prompts for the three images in the flow chart from left to right are as follows: “Please draw an image of a grand piano placed in a concert hall”; “Please draw an image of a student performing on a piano in a concert hall”; “Please draw an image of judges scoring a student performing on a piano in a concert hall”. OpenAI (https://chat.openai.com/chat)(December 20, 2023).

This study employed a within-subjects repeated measures design, encompassing both flow and non-flow conditions for all participants (refer to Figure 1 for the experimental paradigm). Prior to commencing the experiment, subjects received a concise description of “flow”: “*Flow refers to the state in which we are focused on the score and the performance while playing music, and it usually feels like time is passing quickly and other things do not seem to be in your consciousness for a while. It is a very pleasurable experience and is associated with optimal performance.*” The research received ethical approval from the Institutional Review Board of the First People’s Hospital of Qinhuangdao City, and participants provided written informed consent.

During the pre-experimental phase, the researcher conducted a survey to gather demographic information and assess the level of music acquisition, regarded as a measure of skill level in subsequent data analysis, using an independent variable scale. Subsequently, a database of piano repertoire was curated as performance materials, following meticulous screening for repertoire length, time constraints, diversity in composers and their works, and adherence to various structural and stylistic elements. Subjects and judges meeting the experiment’s criteria were recruited through online platforms using web-based methods. The final selection of valid subjects occurred after presenting the structured experimental paradigm and implementing exclusionary inclusion criteria.

Pre-experimental and formal experiments were conducted within the controlled environment of a concert hall, emphasizing strict regulation of experimental conditions. This included standardized piano facilities (utilizing a consistent piano brand, KAWAI, to eliminate potential variations in sound quality) and meticulous control over the physical environment (employing white, cool-colored lighting with a color temperature of 5,000-7000 k; maintaining a temperature range of 20–22 degrees Celsius; and regulating humidity within 40–60%). Subjects were randomly assigned to perform 1 or 2 pieces from the portfolio based on their grade level, presenting a complete piano performance lasting between 20 and 45 min. Post-performance, subjects assessed their own flow state using the CFSS-2, while five experienced music faculty piano majors evaluated their performance, considering factors such as tempo, technique, musicianship, and piece coherence. A follow-up assessment of subjects’ flow state (using the CDFS-2) was conducted one to 2 days after the experiment. Finally, the researcher quantified score information characteristics (e.g., composer, period, style, and notes) in the piano portfolio, aiming to explore the elicitation of flow and its mechanisms on performance when perceived challenge dynamically aligns with the level of musical skill acquisition.

The questionnaires used in the experiment were published and collected in the public student services software of a university in China, and have been verified for their utility in a music performance setting and even validity of the research topic. The data were collected on 18th November 2023.

## 3 Statistical analyses

Statistical analyses conducted using IBM SPSS version 27. To examine the correlation between flow and performance as posited in Hypothesis 1, along with the impact of demographic and musical factors, initial analyses involved descriptive assessments and Pearson’s correlation coefficient tests on the comprehensive dataset. Subsequently, to test Hypothesis 2 and explore the influence of flow on performance within the context of the balance between challenge and skill, sub-model construction was performed on the two variables of challenge and skill.

## 4 Result

### 4.1 Descriptive statistics

#### 4.1.1 Flow and performance scores

To facilitate the examination of the subject group’s flow experience, both state flow and trait flow were separately analyzed descriptively. Table 1 shows that the majority of subjects entered a moderate level of flow, but at a lower level compared to the flow experience in other instrumental studies, validating Moral-Bofill et al. (2023) research. Notably, the overall mean scores for trait flow surpassed those for state flow. Among the individual trait flow dimensions, dimensions 2 to 4 exhibited the highest mean scores, while dimensions 7 to 9 displayed lower mean flow scores, suggesting limited experiences for certain subjects. Additionally, alpha coefficients for both state flow and trait flow, as well as the nine sub-items, were calculated, and all scores exceeded 0.8, indicating good reliability. Furthermore, the mean score for all subjects’ piano performance exceeded 84, indicative of a commendable level of performance (Table 3).

**Table 3:** Descriptive statistics for CFSS-2, CDFS-2 global mean scores, nine sub-dimensions and performance (*N* = 109).

		M	SD	MIN	MAX	α
CFSS-2	F1	3.29	0.92	1.25	5.00	0.88
	F2	3.55	0.89	1.67	5.00	0.81
	F3	3.85	0.90	1.67	5.00	0.87
	F4	4.01	0.81	2.50	5.00	0.87
	F5	3.75	0.94	1.25	5.00	0.88
	F6	3.34	0.97	1.00	5.00	0.88
	F7	3.08	1.02	1.00	5.00	0.84
	F8	3.53	1.00	1.00	5.00	0.83
	F9	3.28	1.08	1.00	5.00	0.91
	Total	3.51	0.75	2.21	5.00	0.96
CDFS-2	F1	3.30	0.73	1.25	5.00	0.85
	F2	3.40	0.77	1.67	5.00	0.82
	F3	3.82	0.83	1.67	5.00	0.86
	F4	3.88	0.73	2.25	5.00	0.90
	F5	3.61	0.76	2.00	5.00	0.91
	F6	3.30	0.78	1.00	5.00	0.82
	F7	2.91	0.86	1.00	5.00	0.82
	F8	3.28	0.92	1.00	5.00	0.85
	F9	3.28	0.89	1.00	5.00	0.89
	Total	12.76	2.17	8.33	18.78	0.95
Performance		84.20	5.21	59.00	95.00	/

M, Mean; SD, Standard deviation; Min, Minimum; Max, maximum; and *α*, Cronbach’s alpha.

#### 4.1.2 Challenges and skills models

The study constructed four balanced challenge and skill models, detailed in Table 4. Given that performance is a key variable, challenge was quantified using performance scores, randomly selecting two subjects each with scores at high and low intermediate levels. Models 1 to 4 represent the absolute difference between SE and the number of years of piano study, average daily practice time, musical ability, and acquired musical skills, respectively, indicating the state of challenge and skill balance. Table 4 reveals that the SE of the piano piece played by Subject No. 1 is the highest. Within the high and low performance levels, the SE of the subject is positively proportional to the score of Model 4, suggesting that flow occurs when the subject’s mastered musical skills maintain a specific equilibrium with the Shannon entropy of the played piece. Additionally, SE represents the complexity of a piece, with higher values indicating greater musical structure complexity. All entropy values are positive within the three performance levels. However, it’s noteworthy that, except for the high-performance group, the entropy values of the middle and low-performance groups are negative. This implies that the complexity of the works in the middle and low-performance groups is less connected to Challenge-Skill Model 4. Further elucidation on the generation of entropy values in the middle and low-performance groups will be provided in the conclusion.

**Table 4:** Challenges and skills model and SE descriptive statistics.

Number	Piece	SE	LSE	RSE	Model 1	Model 2	Model 3	Model 4
1	Three Preludes-for Piano	4.99	3.56	3.77	0.01	0.01	0.01	3.01
2	Barcarolle in F-sharp Major Op.60	4.59	3.44	3.97	0.41	1.41	0.41	2.41
3	Etude in F minor	4.20	2.74	3.31	−2.20	−0.20	−1.20	2.80
4	Nocturne in E flat major	4.83	4.29	3.68	−2.83	1.17	−1.83	−1.83
5	Czerny 740 No.3	4.59	3.32	4.06	−0.59	−2.59	−0.59	3.41
6	Inventions et Sinfonias	4.38	2.98	3.10	−2.38	−2.38	−2.38	−1.38

Number is the serial number of the subjects, from 1 to 6 representing two subjects at the high school and low school levels of piano performance; piece is the name of the musical work performed by the subject; SE is Shannon Entropy; LSE is the left hand SE of the work, Left hand SE; and RSE is the Right hand SE of the work, Right Hand SE (See appendix for more information on piano works).

### 4.2 Relevant relationship

#### 4.2.1 Flow and performance

**Figure 2:**
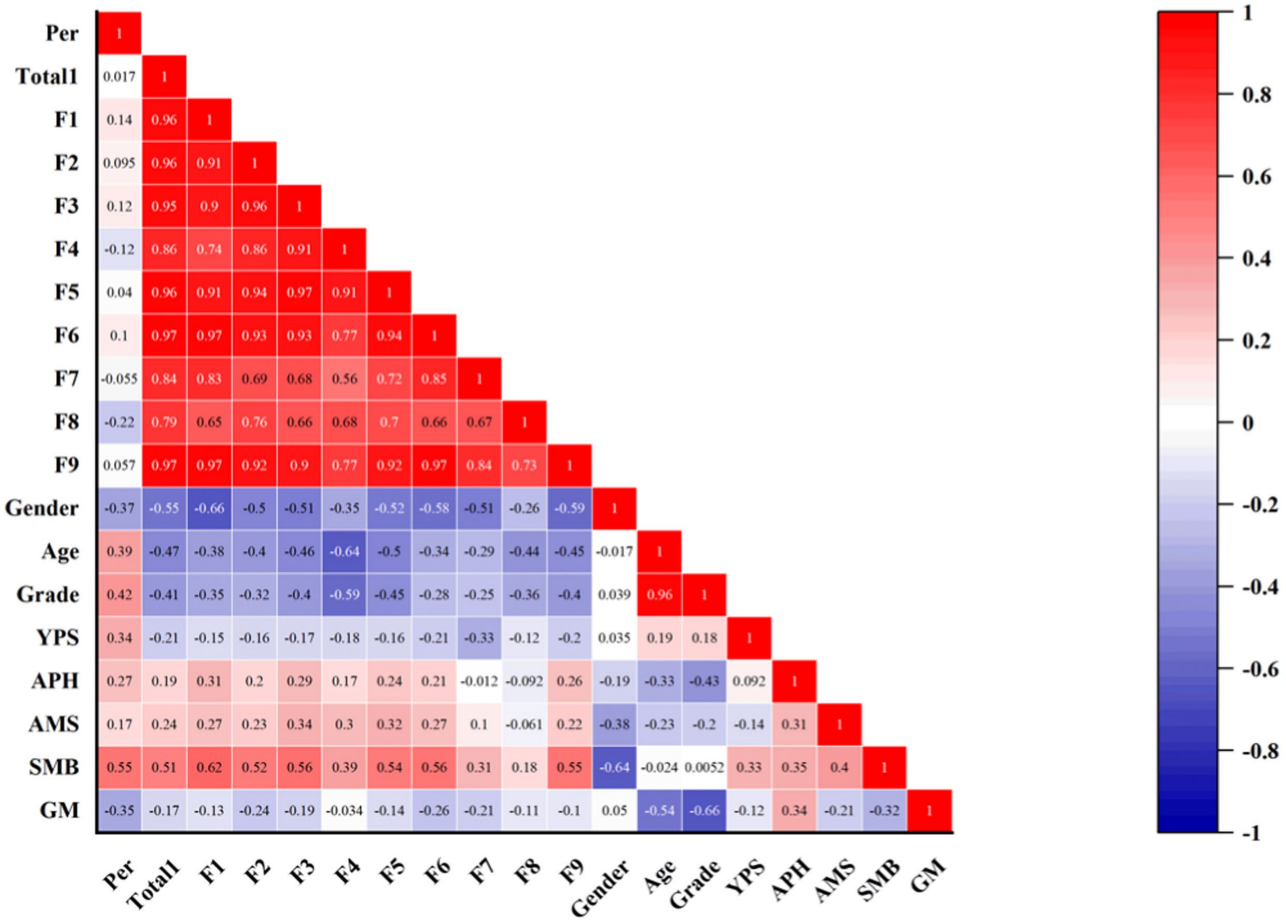
Results of CFSS-2, demographic, and performance-related analyses. Per: performance; Total1: total mean score of CFSS-2; F1-F9: average scores on nine dimensions; Genders, Ages, and Grades are not abbreviated; YPS, years of piano study; AHP, Average hours of piano practice per day; AMS, Acquired musical skills; SMB, Self-assessment of musical ability; GM, Genre of music.

Figure 2 shows the intercorrelation between the study variables of state flow and performance, according to the CFSS-2, indicating that there is a positive correlation between flow and performance *r* = 0.236*, *p* < 0.05, with dimension 1 *r* = 0.277**, *p* < 0.01, dimension 3 *r* = 0.305**, *p* < 0.01, and dimension 6 *r* = 0.247**, *p* < 0.01 being significantly positively correlated with performance. Among the demographic factors, the results showed a significant positive correlation between subjects’ age *r* = 0.271**, *p* < 0.01, grade *r* = 0.344**, *p* < 0.01 and performance, but there was no correlation between gender and performance *r* = −0.097, *p* < 0.01. In addition, there was a significant positive correlation between the number of years of musical training received by the subjects and performance *r* = 0.287**, *p* <. 01, and the average number of hours of musical training per day likewise had a significant positive correlation with performance *r* = 0.312**, *p* < 0.01. Surprisingly, there was no correlation between the musical skills acquired by the subjects and performance *r* = 0.166, *p* < 0.01. However, musical ability had a significant positive correlation with performance *r* = 0.441**, *p* < 0.01.

**Figure 3:**
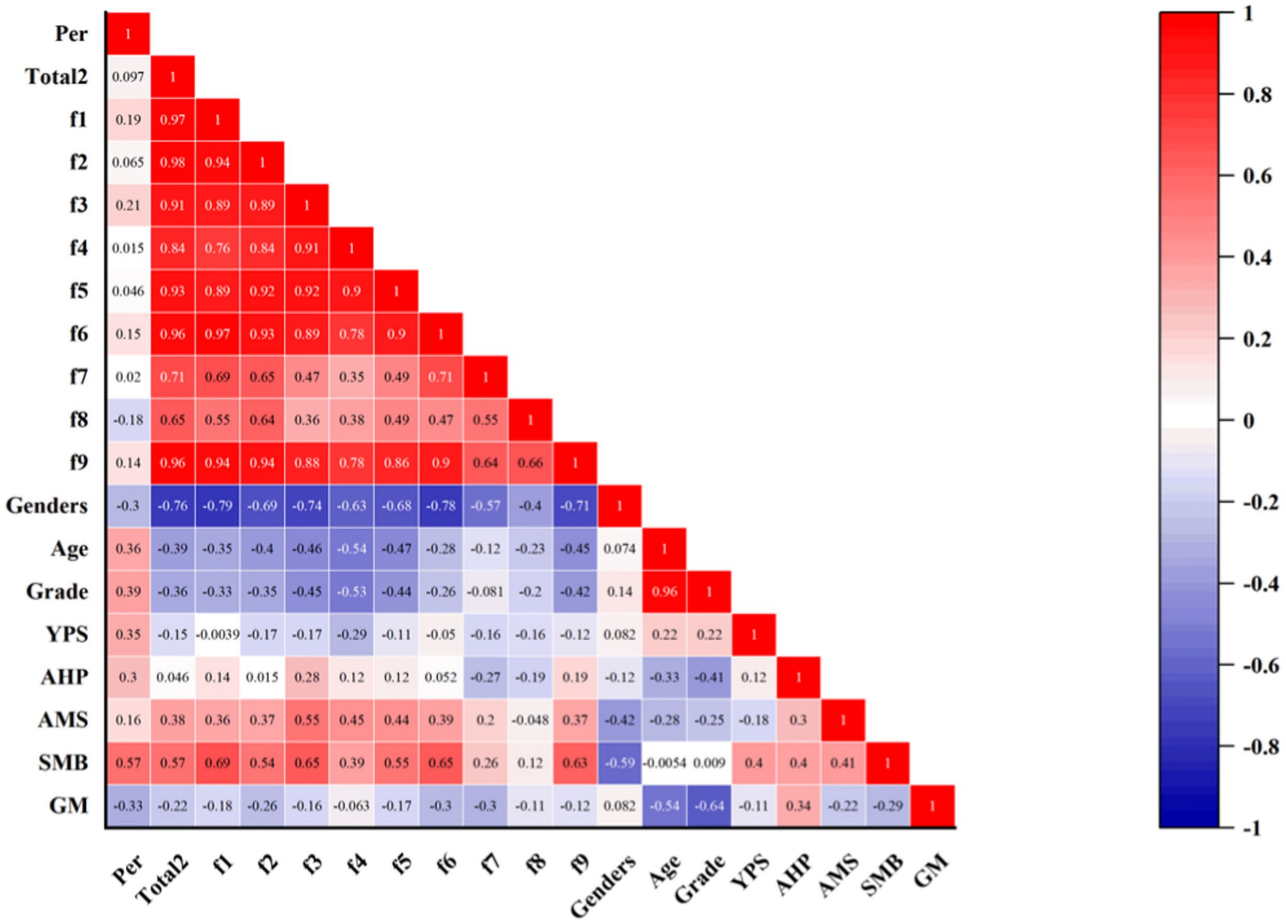
Results of CDFS-2, demographic, and performance-related analyses. Total2: CDFS-2 average score; the rest of the abbreviations are consistent with Figure 2.

Figure 3 shows the intercorrelations between the study variables of trait flow on performance, according to the CDFS-2, indicating that there is a significant positive correlation between flow and performance *r* = 0.268**, *p* < 0.01, with Dimension 1 *r* = 0.300**, *p* < 0.01, Dimension 3 *r* = 0.291**, *p* < 0.01, and Dimension 9 *r* = 0.294**, *p* < 0.01 being significantly positively correlated with performance. The results for demographic factors and performance were consistent with the results for state flow, with age and grade significantly and positively correlated with performance *r* = 0.271**, *p* < 0.01, *r* = 0.344**, *p* < 0.01, and the results for gender were similarly uncorrelated with performance *r* = −0.097, *p* < 0.01. Additionally, the number of years that the subjects had received music training, the average number of hours of music training per day, and the musical abilities and performance were consistent with the results for state mobility, all of which were significantly positively correlated *r* = 0.287**, *p* < 0.01, *r* = 0.312**, *p* < 0.01, *r* = 0.441**, *p* < 0.01, and there was no correlation between acquired musical skills and performance *r* = 0.166, *p* < 0.01.

#### 4.2.2 The relationship between flow and performance in the balance of challenges and skills

**Figure 4:**
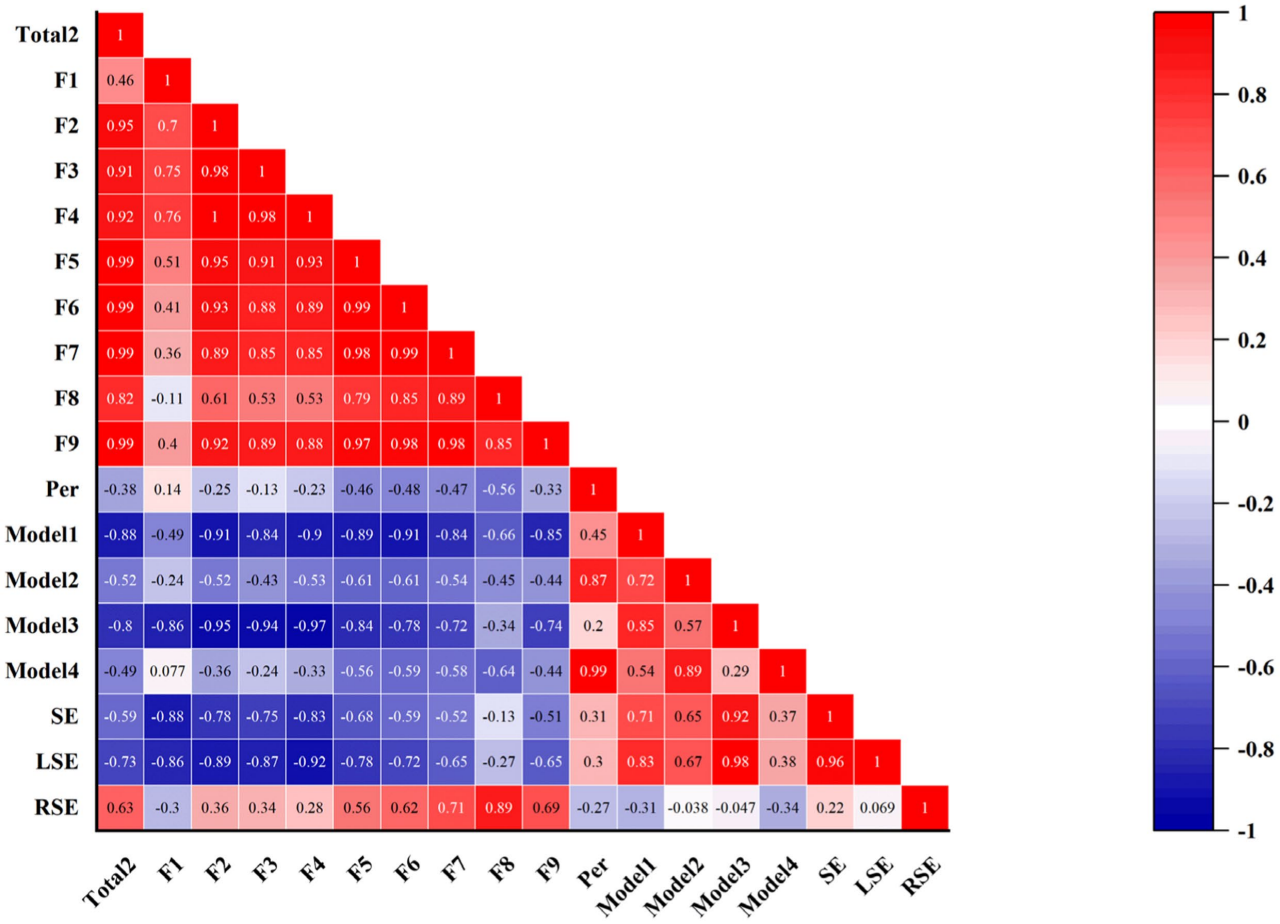
Results of CDFS-2, Performance and Challenge Skills Model, and SE Correlation Analysis. Total2: CDFS-2 average score; F1-F9: average scores on nine dimensions; Model 1 - Model 4: Balance of Challenge and Skills model constructed in 4.1.3, 4 in total. SE, Shannon Entropy; LSE, Left Hand SE; RSE, Right Hand SE.

The Challenge-Skills Balance dimension, as an important but not exclusive factor in the production of the flow experience, contains the following question items: *q1 just now I was challenged but I believe that my skills are able to cope with this challenge, q8 just now my abilities matched the high demands of the situation, q17, just now I felt that my abilities were sufficient to meet the high demands of the situation, q25 just now both the challenge and my skills were at an equally high level*. Based on the above descriptive analyses, we found that the dimension did not score the highest in the state flow versus the trait flow; the correlation between the dimension and the total flow score in both flows was more significant in the trait flow *r* = 0.870**, *p* < 0.01, and the same finding was obtained in the correlation with performance, which was more relevant in the trait flow *r* = 0.300**, *p* < 0.01. Therefore, we explored the correlation between the challenge and the Skills model in the trait flow by correlating the model with the quantified Challenge and Skills, respectively, with the trait flow as shown in Figure 4. The results showed that none of the four models set up according to the above were correlated with the total mean score of the flow, but there was a certain negative correlation between Model 3 and Dimension 4 of the trait flow *r* = −0.860*, *p* < 0.05, and in the correlation with Performance, the results showed that there was a positive Performance and Model 2 correlation *r* = 0.870*, *p* < 0.05, and a significant positive correlation with Model 4 *r* = 0.996*, *p* < 0.01, but the result was not found for Challenge and Skills within the model. Surprisingly, in the challenge dimension, the right-hand SE of the piano piece was positively correlated with dimension 7 *r* = 0.836*, *p* < 0.05, dimension 8 *r* = 0.856*, *p* < 0.05, and dimension 9 *r* = 0.825*, *p* < 0.05, in trait flow. In addition, in the challenge, Model 3 was positively correlated with left-handed SE *r* = 0.905*, *p* < 0.05. No correlations existed between the other main variables relevant to this study.

## 5 Discussion

The present study firstly validated the pattern of flow state attainment and its evoked mechanisms in Chinese music college students in a piano performance situation, and compared these results with the findings of Jha et al. (2022). Subjects in the study demonstrated the same findings, all being able to achieve a flow experience at a level where challenge and skill were matched to each other. This finding demonstrates the cross-cultural validity of the flow experience.

By quantifying music ontology data and music acquisition levels, we have successfully established an access framework in equilibrium, utilizing challenge and skill as the metrics for flow. The equilibrium level is represented by the quantification of the relationship between the complexity of the work, characterized by SE, and the degree of musical skills acquired by the subject. According to our findings, in the high-performance level, the SE value of Challenge and Skill Model 4 is significantly correlated with the growing complexity of the work as musical skill increases. This implies that subjects are more likely to experience flow when Model 4 is positively proportional to the complexity of the work. In contrast, in the middle and low-performance levels, some SE values yielded negative numbers, suggesting that complexity cannot be accurately measured by SE values in piano practice pieces. Consequently, establishing a conclusive relationship between the challenge-skill model and entropy values was challenging, requiring further research. This study marks the first quantification of this equilibrium relationship in the exploration of flow theory in the field of music performance, with implications for subsequent research. Specifically, self-reported levels of music acquisition prove to be valid skill guidelines. The results reported in this paper demonstrate that the SE algorithm effectively quantifies the complexity of musical compositions in a well-documented manner.

In addition to this, external factors such as age, grade, years of musical training, average daily musical training time, and musical ability could also partially elucidate the induction of flow, with grade and musical ability potentially emerging as more influential factors in this regard. These findings align with and reinforce the conclusions drawn by Cohen and Bodner (2019). Notably, the current study revealed novel insights from music-related information, emphasizing the significant impact of musical ability on flow experiences, which progressively shapes the unfolding experiences in response to outcomes within the flow experience (Marin and Bhattacharya, 2013). For instance, as grade level rises, there is a discernible impact on frequency (Koehn and Morris, 2014) versus duration, among other factors – a result that awaits further validation. Contrary to expectations, indicators of gender and acquired musical skills proved less explanatory in the emergence of flow, possibly attributed to the geo-cultural nature of the sample in this paper.

Equally pivotal in addressing the second research question of this paper, a notable positive correlation emerged between the attainment of a flow state and piano performance. Furthermore, the impact of flow, induced by the quantified challenge-skill balance model, exhibited a more pronounced effect in high-performance scenarios. Here, SE in Music, serving as a quantitative representation of the musical characteristics inherent in challenge and skill, played a moderating role in the connection between flow and performance. This result provides further evidence of the important impact of the balanced modeling of challenge and skill in the flow experience on the enhancement of musical ability in a competitive environment (Bullerjahn et al., 2020). Changes in SE, a key aspect of characterizing the complexity of a piano piece (Ribeiro et al., 2012; Tozman et al., 2017), demonstrated a relational effect on performance flow, as evidenced in the results (Marty-Dugas et al., 2021; Tan et al., 2021). In summary, the dynamic equilibrium between challenge and skill, identified as a significant influencer of flow, yielded unexpected outcomes in attempts to quantify the complexity of a work. Simultaneously, this approach has introduced novel perspectives for interpreting the intricate relationship between flow and performance (Zielke et al., 2023).

## 6 Limitation

While diligent efforts have been exerted to regulate experimentally relevant variables, it is crucial to acknowledge potential limitations in the generalizability of these findings, considering regional disparities, instrument-specific nuances, and the distinctive experimental context. Consequently, the scope of these results may be constrained within the realm of piano performance and may not be universally applicable across diverse musical domains. There are potential limitations to the present study’s induced modeling of the flow experience and the use of SE as one of the objective measures of piano challenge, due to the fact that there are many other unpredictable factors that contribute to the quantitative limitations of the challenge during a true challenge, and, also, due to the pianist’s respective playing skills. Thus, future research could start with objective quantification of musical compositions (e.g., SE) and explore other measures of challenge in the flow experience. Additionally, the relationship between areas other than the field of piano performance (e.g., other instruments versus the experience of flow in a musical listening environment) and SE should be investigated, suggesting an expansion of the current theme. Moreover, cross-sectional investigations of this nature may encounter unforeseen factors influencing the emergence of the flow experience. Future research endeavors should explore connections beyond piano performance, such as diverse musical instruments or the encounter of flow in a musical listening environment, highlighting an expansion of the present inquiry. It is imperative to conduct further studies to authenticate the aforementioned results, fostering a comprehensive understanding of the intricate relationship between flow and musical attributes. Additionally, the correlation between the physical attributes of music and the flow state remains an unanswered query, prompting subsequent research to incorporate these factors into the investigative framework.

Subsequent research endeavors could delve into a detailed exploration of specific underlying factors. Examining these features in depth may provide a more nuanced understanding of the intrinsic dynamic characteristics associated with positive emotions and flow. Such insights, in turn, could serve as a means for enhancing well-being through musical engagement. In summary, additional research in the field of music practice is essential to assist less experienced students in cultivating flow experiences during their practice sessions, ultimately fostering more meaningful experiences in the realm of music learning.

## Data availability statement

The original contributions presented in the study are included in the article/Supplementary material, further inquiries can be directed to the corresponding author/s.

## Ethics statement

The studies involving humans were approved by the First People’s Hospital of Qinhuangdao City. The studies were conducted in accordance with the local legislation and institutional requirements. The participants provided their written informed consent to participate in this study.

## Author contributions

JL: Methodology, Funding acquisition, Writing – original draft. YX: Writing – review & editing. XW: Validation, Writing – review & editing. XY: Writing – original draft, Visualization. ShL: Writing – original draft, Resources. ML: Writing – original draft, Validation. XR: Project administration, Writing – original draft. DY: Supervision, Investigation, Writing – original draft. SiL: Visualization, Writing – original draft. ZJ: Writing – original draft, Data curation. QG: Writing – original draft, Resources. MY: Data curation, Writing – original draft. QX: Writing – review & editing, Supervision, Data curation.
