# Supplementary material for: Flow and performance: a quantitative study of elicitation modeling in a piano performance perspective
Source: Front Psychol. 2024 Jul 15;15:1386831. doi: 10.3389/fpsyg.2024.1386831 (PMC11285101; doi:10.3389/fpsyg.2024.1386831)
Supplement: Supplementary file 1 [file Data_Sheet_1.zip › 1386831_SupMaterial/138631_Xu_Table_2.docx]

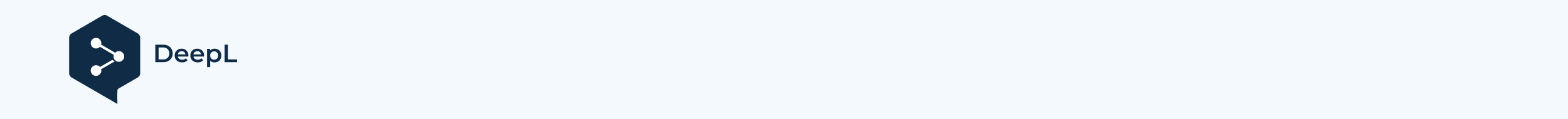
Flow of State Scale-2 (CFSS-2)

Subscribe to DeepL Pro to edit this document.
Visit [www.DeepL.com/pro](https://www.deepl.com/pro?cta=edit-document) for more information.

Dear Participants: Hello! I am a researcher from the MUAI Institute of Music Science and Artificial Intelligence at Yanshan University, and this questionnaire will be used for the project "Flow in the Piano". For information on the latest progress of the experiment, please follow the MUAI public number!
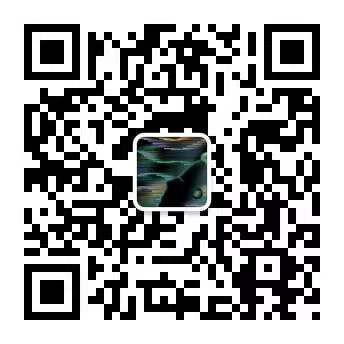


This questionnaire consists of 33 questions and is expected to take 5-10 minutes! Thank you very much for your support and cooperation! All the data obtained from this questionnaire will be used for experimental research. We assure you that we will not leak any of your information to third parties, and we will keep your personal privacy completely confidential! If you have any questions and suggestions, please contact Please contact email：208989341@qq.com Now let's get started right away!

I. Basic information

1. Name [fill in the blank]

________________________

2. Grades 【One-for-one】

○ freshman

○ sophomore

○ junior student

○ senior student

3、Gender 【Optional】

○ male

○ Female

4. Age 【Optional】

○ 18 years old

○ 19 years old

○ 20 years old

○ 21 years old

○ 22 years old

○ 23 years old

○ 23 years or older

5. Years of piano study 【Optional questions

○ 1-3 years

○ 4-6 years

○ 7-9 years

○ 10-13 years

○ 14-16

○ 17-19 years

○ More than 20 years

6. Average length of piano practice per day [Single-choice question

○ Within 30 minutes

○ 30 minutes - 1 hour

○ 1 hour-1.5 hours

○ 1.5 hours - 2 hours

○ 2 hours - 3 hours

○ 4 hours - 6 hours

○ 5 hours - 7 hours

7、What are the musical skills you have acquired? [Multiple choice questions

□ vocal performance

□ instrumental performance

□ music appreciation

□ harmony theory knowledge

□ music score readability

□ solfeggio

□ music composition

□ music history knowledge

□ fundamental knowledge of music theory

□ None

8. Do you have any health condition at present? If yes, please explain in detail 【Optional】.

○ Yes ○ None

9、Your assessment of your own musical ability： 【Evaluation Question】 (Please fill in 1-5 numbers to score)

Score _____

Tagged _____

II. The Flow of State Scale-2 (CFSS-2)

Please answer the following questions based on your experience during the piano exam you just completed. These questions relate to a variety of thoughts and feelings you may have experienced during the examination you have just completed, and there are no right or wrong answers. Think about how you felt during the course of the exam process and then answer the questionnaire using the hierarchy below. Each question is labeled with the number that best matches your experience.

The scale is as follows: 1----"Totally Disagree" 2----"Disagree" 3----"Neither Agree nor Disagree" 4----"Oppose" 5----"Totally Agree "Disagree" 5---- "Totally agree"

During the exam:

10、I have just been challenged, but I am confident that my skills will be able to meet this challenge. 【Scoring Question】 (Please fill in the numbers 1-5 to score)

1 is totally disagree,5 is totally agree, Your rating is ____

11、Just now I know exactly what I want to do. 【Scoring Question】 (Please fill in numbers 1-5 to score)

1 is totally disagree,5 is totally agree, Your rating is ____

12. Just now I did have a good idea of how well I was doing. 【Scoring Question】 (Please fill in the numbers from 1 to 5 to score)

1 is totally disagree,5 is totally agree, Your rating is ____

13, Just now my attention was fully focused on the activity that was going on. 【Scoring Question】 (Please fill in the numbers from 1 to 5 to score)

1 is totally disagree,5 is totally agree, Your rating is ____

14, Just now I don't care how others might see me. 【Scoring Question】 (Please fill in the numbers 1-5 to score)

1 is totally disagree,5 is totally agree, Your rating is ____

15, Just now time seems to have changed (either slowed down or sped up). 【Scoring Question】 (Please fill in the numbers 1-5 to score)

1 is totally disagree,5 is totally agree, Your rating is ____

16, Just now I really enjoyed that experience. 【Scoring Question】 (Please fill in numbers 1-5 to score)

1 is totally disagree,5 is totally agree, Your rating is ____

17, Just now my ability to match the high demands of the situation. 【Scoring Question】 (Please fill in the numbers from 1 to 5 to score)

1 is totally disagree,5 is totally agree, Your rating is ____

18, Just now the action seemed to happen naturally. [Scoring Question] (Please fill in the numbers from 1 to 5 to score)

1 is totally disagree,5 is totally agree, Your rating is ____

19, Just now I realized clearly what I wanted to do. 【Scoring Question】 (Please fill in the numbers 1-5 to score)

1 is totally disagree,5 is totally agree, Your rating is ____

20. Just now I know how well I did. 【Scoring Question】 (Please fill in the numbers from 1 to 5 to score)

1 is totally disagree,5 is totally agree, Your rating is ____

21、Just now I can effortlessly is my attention on the ongoing activities. 【Scoring Question】 (Please fill in the numbers 1-5 to score)

1 is totally disagree,5 is totally agree, Your rating is ____

22. Just now I felt that I was able to control the activity that was going on. 【Scoring Question】 (Please fill in the numbers from 1 to 5 to score)

1 is totally disagree,5 is totally agree, Your rating is ____

23, Just now I don't care what others might say about themselves. 【Scoring Question】 (Please fill in the numbers 1-5 to score)

1 is totally disagree,5 is totally agree, Your rating is ____

24, Time just passed differently than usual. 【Scoring Question】 (Please fill in the numbers 1-5 to score)

1 is totally disagree,5 is totally agree, Your rating is ____

25、Just now I love the feeling of completing an action and want to experience it again. 【Scoring Question】 (Please fill in numbers 1-5 to score)

1 is totally disagree,5 is totally agree, Your rating is ____

26、Just now I feel that I am competent enough to meet the high demands of the situation. 【Scoring Question】 (Please fill in the numbers from 1 to 5 to score)

1 is totally disagree,5 is totally agree, Your rating is ____

27, I just automated my movements and didn't think too much about it. 【Scoring Question】 (Please fill in numbers 1-5 to score)

1 is totally disagree,5 is totally agree, Your rating is ____

28. When I just finished the action, I knew exactly how well I was doing. 【Scoring Question】 (Please fill in the numbers from 1 to 5 to score)

1 is totally disagree,5 is totally agree, Your rating is ____

29、Just now I am totally focused. 【Scoring Question】 (Please fill in the numbers 1-5 to score)

1 is totally disagree,5 is totally agree, Your rating is ____

30. Just now I had a sense of complete control. 【Scoring Question】 (Please fill in the numbers 1-5 to score)

1 is totally disagree,5 is totally agree, Your rating is ____

31, Just now I don't care how well I perform. 【Scoring Question】 (Please fill in the numbers 1-5 to score)

1 is totally disagree,5 is totally agree, Your rating is ____

32、Just now I feel that time is passing faster than usual. 【Scoring Question】 (Please fill in the numbers 1-5 to score)

1 is totally disagree,5 is totally agree, Your rating is ____

33, That experience just made me feel ecstatic. 【Scoring Question】 (Please fill in the numbers 1-5 to score)

1 is totally disagree,5 is totally agree, Your rating is ____

34, just challenged and my skills are at an equally high level. 【Scoring Question】 (Please fill in the numbers 1-5 to score)

1 is totally disagree,5 is totally agree, Your rating is ____

35、Just now I acted out of instinct and automatically without having to think. 【Scoring Question】 (Please fill in the numbers 1-5 to score)

1 is totally disagree,5 is totally agree, Your rating is ____

36, Just now my goals were clearly defined. [Scoring Question] (Please fill in the numbers from 1 to 5 to score)

1 is totally disagree,5 is totally agree, Your rating is ____

37, Just now I was able to judge how well I was doing based on the action being completed. [Scoring Question] (Please fill in the numbers from 1 to 5 to score)

1 is totally disagree,5 is totally agree, Your rating is ____

38, Just now I was fully focused on the task at that moment. 【Scoring Question】 (Please fill in the numbers 1-5 to score)

1 is totally disagree,5 is totally agree, Your rating is ____

39, Just now I felt in complete control of my body. 【Scoring Question】 (Please fill in the numbers from 1 to 5 to score)

1 is totally disagree,5 is totally agree, Your rating is ____

40, Just now I don't worry about how others might perceive me. 【Scoring Question】 (Please fill in the numbers from 1 to 5 to score)

1 is totally disagree,5 is totally agree, Your rating is ____

41. I just lost my normal sense of time. 【Scoring Question】 (Please fill in the numbers 1-5 to score)

1 is totally disagree,5 is totally agree, Your rating is ____

42, Just now I realized that that experience is one of the best rewards. 【Scoring Question】 (Please fill in numbers 1-5 to score)

1 is totally disagree,5 is totally agree, Your rating is ____
