# Supplementary material for: Flow and performance: a quantitative study of elicitation modeling in a piano performance perspective
Source: Front Psychol. 2024 Jul 15;15:1386831. doi: 10.3389/fpsyg.2024.1386831 (PMC11285101; doi:10.3389/fpsyg.2024.1386831)
Supplement: Supplementary file 1 [file Data_Sheet_1.zip › 1386831_SupMaterial/138631_Xu_Table_1.docx]

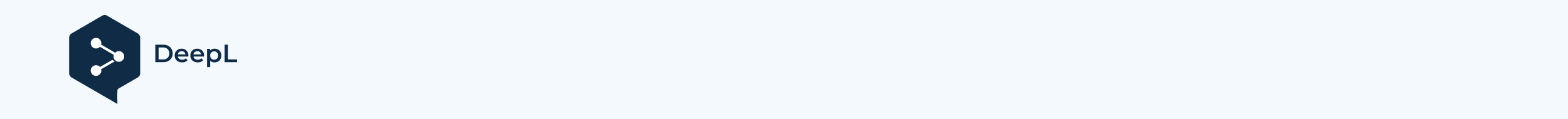
Trait Flow Scale-2 (CDFS-2)

Subscribe to DeepL Pro to edit this document.
Visit [www.DeepL.com/pro](https://www.deepl.com/pro?cta=edit-document) for more information.

Dear Participants: Hello! I am a researcher from the MUAI Institute of Music Science and Artificial Intelligence at Yanshan University, and this questionnaire will be used for the project " Flow in the Piano". For the latest information about the progress of the experiment, welcome to follow the MUAI public number!
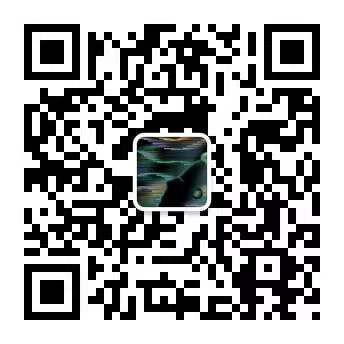


This questionnaire consists of 33 questions and is expected to take 5-10 minutes! Thank you very much for your support and cooperation! All the data obtained from this questionnaire will be used for experimental research. We assure you that we will not leak any of your information to third parties, and we will keep your personal privacy completely confidential!

If you have any questions and suggestions, please contact

Please contact email 208989341@qq.com Let's get started right away!

I. Basic information

1. Name [fill in the blank]

________________________

2. Grades 【One-for-one】

○ freshman

○ sophomore

○ junior student

○ senior student

3、Contact information 【Fill in the blank

________________________

II. Trait Flow Scale-2 (CDFS-2)

Please answer the following questions based on your experience in the piano performance exam. These questions relate to thoughts and feelings you may have experienced while participating in the examination process. It is possible that you may have sometimes, always, or never experienced these characteristics. There are no right or wrong answers. Thinking back to how often you experienced each characteristic during the exam, choose the number that best matches your experience.

The hierarchy is as follows: 1----"Never" 2----"Rarely" 3----"Sometimes" 4----"Often " 5----"always"

When involved in a piano performance exam:

4. I have encountered a challenge, but I am confident that my skills will be able to cope with this challenge. 【Scoring Question】 (Please fill in the numbers from 1 to 5 to score)

With 1 being never and 5 being always, your rating is ____

5. I know exactly what I want to do. 【Scoring Question】 (Please fill in the numbers from 1 to 5 to score)

With 1 being never and 5 being always, your rating is ____

6. I do have a good idea of how well I am doing. 【Scoring Question】 (Please fill in the numbers from 1 to 5 to score)

With 1 being never and 5 being always, your rating is ____

7, My attention is fully focused on the activity that is going on. [Scoring Question] (Please fill in the numbers 1-5 to score)

With 1 being never and 5 being always, your rating is ____

8. I don't care what others may think of me. 【Scoring Question】 (Please fill in the numbers from 1 to 5 to score)

With 1 being never and 5 being always, your rating is ____

9. Time seems to have changed (either slowed down or sped up). [Scoring Question] (Please fill in the numbers 1-5 to score)

With 1 being never and 5 being always, your rating is ____

10. I really enjoyed the experience. 【Scoring Question】 (Please fill in numbers 1-5 to score)

With 1 being never and 5 being always, your rating is ____

11. My ability to match the high demands of the situation. [Scoring Question] (Please fill in the numbers from 1 to 5 to score)

With 1 being never and 5 being always, your rating is ____

12. Actions seem to happen naturally. [Scoring Question] (Please fill in the numbers from 1 to 5 to score)

With 1 being never and 5 being always, your rating is ____

13. I realize clearly what I want to do. 【Scoring Question】 (Please fill in the numbers from 1 to 5 to score)

With 1 being never and 5 being always, your rating is ____

14. I know how well I am doing. [Scoring Question] (Please fill in the numbers from 1 to 5 to score)

With 1 being never and 5 being always, your rating is ____

15、I can effortlessly is my attention on the ongoing activities. 【Scoring Question】 (Please fill in the numbers 1-5 to score)

With 1 being never and 5 being always, your rating is ____

16. I feel in control of the activities that are going on. [Scoring Question] (Please fill in the numbers from 1 to 5 to score)

With 1 being never and 5 being always, your rating is ____

17, I don't care what others may think of me. 【Scoring Question】 (Please fill in the numbers from 1 to 5 to score)

With 1 being never and 5 being always, your rating is ____

18. Time passes differently than usual. 【Scoring Question】 (Please fill in the numbers 1-5 to score)

With 1 being never and 5 being always, your rating is ____

19、I love the feeling of completing an action and want to experience it again. 【Scoring Question】 (Please fill in the numbers 1-5 to score)

With 1 being never and 5 being always, your rating is ____

20. I feel that I am competent enough to meet the high demands of the situation. [Scoring Question] (Please fill in the numbers from 1 to 5 to score)

With 1 being never and 5 being always, your rating is ____

21. I automated my movements and didn't think too much about it. 【Scoring Question】 (Please fill in numbers 1-5 to score)

With 1 being never and 5 being always, your rating is ____

22. When completing an action, I know exactly how well I did. 【Scoring Question】 (Please fill in numbers 1-5 to score)

With 1 being never and 5 being always, your rating is ____

23, I am totally focused. 【Scoring Question】 (Please fill in numbers 1-5 to score)

With 1 being never and 5 being always, your rating is ____

24. I have a sense of complete control. 【Scoring Question】 (Please fill in the numbers from 1 to 5 to score)

With 1 being never and 5 being always, your rating is ____

25. I don't care how well I perform. [Scoring Question] (Please fill in the numbers 1-5 to score)

With 1 being never and 5 being always, your rating is ____

26、I feel that time passes faster than usual. 【Scoring Question】 (Please fill in the numbers 1-5 to score)

With 1 being never and 5 being always, your rating is ____

27. That experience made me feel ecstatic. 【Scoring Question】 (Please fill in the numbers 1-5 to score)

With 1 being never and 5 being always, your rating is ____

28, The challenge and my skills are at an equally high level. 【Scoring Question】 (Please fill in numbers 1-5 to score)

With 1 being never and 5 being always, your rating is ____

29、I act out of instinct and automatically without having to think. 【Scoring Question】 (Please fill in the numbers 1-5 to score)

With 1 being never and 5 being always, your rating is ____

30. My goals are clearly defined. [Scoring Question] (Please fill in the numbers from 1 to 5 to score)

With 1 being never and 5 being always, your rating is ____

31. I am able to judge how well I am doing based on the action being completed. [Scoring Question] (Please fill in the numbers from 1 to 5 to score)

With 1 being never and 5 being always, your rating is ____

32, I am fully focused on the task at hand. 【Scoring Question】 (Please fill in numbers 1-5 to score)

With 1 being never and 5 being always, your rating is ____

33. I feel in complete control of my body. 【Scoring Question】 (Please fill in the numbers from 1 to 5 to score)

With 1 being never and 5 being always, your rating is ____

34, I don't worry about how others might perceive me. 【Scoring Question】 (Please fill in the numbers from 1 to 5 to score)

With 1 being never and 5 being always, your rating is ____

35. I lost my normal sense of time. [Scoring Question] (Please fill in the numbers 1-5 to score)

With 1 being never and 5 being always, your rating is ____

36, This experience is one of the best rewards. [Scoring Question] (Please fill in the numbers 1-5 to score)

With 1 being never and 5 being always, your rating is ____

End of Questionnaire Filling ~ Thank you very much for your cooperation with us this time!
